# Healthcare Disparity and Comorbidity Burden in Heart Failure Patients Over the Age of 80

**DOI:** 10.1093/geroni/igab046.2386

**Published:** 2021-12-17

**Authors:** Anna Blach, Amanda Pangle, Jeanne Wei, Gohar Azhar

**Affiliations:** 1 University of Arkansas for Medical Sciences, Little Rock, Arkansas, United States; 2 UAMS, UAMS, Arkansas, United States

## Abstract

The healthcare industry is currently struggling with providing access and coverage for a rapidly ageing and increasingly diverse population with multiple co-morbid conditions. This retrospective study analyzed the electronic health records of elderly heart failure patients (age range 80-103; mean 87 ±4.9) for common co-morbid conditions of hypertension, hyperlipidemia, dementia and diabetes mellitus. Chart review analysis of 316 patients showed a racial distribution of 251 White vs. 65 Black patients (79% vs. 21%). Male patients were under-represented (B= 13.8% and W= 26.3%). Females patients predominated (B= 86.2% and W= 73.7%). Overall, the prevalence of all four comorbidities was approximately three times higher in Blacks (18.5%) vs. White (7.2%). The proportion of Blacks and Whites with HTN and was comparable at 98.5 and 92.4% respectively. Hyperlipidemia was present in 84.6% Black and 63.3% White. The diagnosis of diabetes was higher in Blacks, 41.5% compared to Whites, 21.9%. The greatest disparity was in the diagnosis of dementia which was higher in Blacks, 61.5% vs Whites, 44.6%. Our study is unique for studying healthcare disparity in octogenarian and nonagenarian residing in a rural setting. Our results also highlight the importance of making a special effort to engage older Black patients in seeking healthcare in addition to designing strategies to reduce barriers that impede access and availability of resources and clinical care, especially in economically underserved regions of the country.

